# Psychiatric reform in Greece: an overview

**DOI:** 10.1192/pb.bp.116.053652

**Published:** 2016-12

**Authors:** George Giannakopoulos, Dimitris C. Anagnostopoulos

**Affiliations:** 1 Department of Child Psychiatry, School of Medicine, National and Kapodistrian University of Athens, Athens, Greece

## Abstract

Leros became infamous worldwide in the 1980s because of a scandal in its mental institution, the Leros asylum. The scandal provoked universal outrage and the international pressure triggered the Greek mental health reform. Under the reform projects Leros I and Leros II (1990–1994), numerous interventions took place in the Leros asylum as part of deinstitutionalisation. Following that, the Psychargos programme advanced developments for community-based services. Deinstitutionalisation and development of community mental health services have advanced significantly since the 1980s. However, this reform is still incomplete, given that sectorisation, adequate primary care policies, inter-sectoral coordination and specialised services are under-developed. This problematic situation is further complicated by the severe impact of the current financial crisis.

Leros, one of the Greek islands in the Aegean Sea, has become infamous worldwide because of its mental institution, which provoked universal outrage in the 1980s. The Leros asylum was established in 1958, taking the official name Colony for Psychopaths, in order to disperse residents of other psychiatric asylums in Greece. In a country that was experiencing the disastrous aftermath of World War II and the Civil War, socioeconomic and political circumstances created conditions for the reinforcement of the asylum structure and a dramatic increase in the number of institutionalised patients in public mental hospitals. The asylum grounds had been used as Italian naval and air force bases during the 1920s and 1930s and later they were used by the Greek state as military camps, boarding schools for orphaned children and offspring of Greek Civil War refugees, and political prisoner camps. Most patients were transferred to Leros *en masse* from other asylums without prior notice. In total, 4000 psychiatric patients were moved, with the most substantial transfer of 900 people in 1965. The referrals to Leros were either with the consent of mental health professionals or covered up. The number of patients institutionalised in Leros increased disproportionately to the available infrastructure and medical, nursing and ward staff. In 1982 all referrals to Leros stopped.^1,2^

The post-dictatorship political situation following the Greek military junta rule of 1967–1974 signalled innovative democratising changes in tertiary education and in medical and psychiatric training as well as the healthcare system overall. During that time prevention-focused medical specialties, such as social medicine and child and adolescent psychiatry, emerged. At the same time, progressively thinking medical doctors struggled towards establishing a public national health system in Greece. These factors made a substantial contribution to the shift from a biomedical to a biopsychosocial approach to health and the need for community-based healthcare.^3^ The National Health System Law 1397 passed in 1983 laid the basis for a new model of psychiatric service provision. The imperative need for change in psychiatry was reflected in the Greek psychiatric reform introduced in 1984 with the European Community Regulation 815. The reform aimed at training mental health professionals, creating a decentralised community network of preventive, specialised treatment and rehabilitation services, deinstitutionalising psychiatric patients with chronic conditions and reducing admissions to mental hospitals. However, until the 1990s the reform process was mainly characterised by non-absorption of European Union (EU) funds and stagnation.^4^ In 1989, the UK *Observer* published an article on Leros, presenting the appalling conditions in which the patients lived there. Residents suffered severe deprivation, extreme institutionalisation and violation of basic human rights. International pressure on the asylum then escalated and mental health professionals called for immediate action. Eventually, it was the Leros case that triggered the process of reform of Greek mental health institutions.^5^

Under the two reform projects, Leros I and Leros II (1990–1994), numerous interventions took place. In 1990, 19 community hostels were established on Leros. The majority of patients were discharged from the asylum in 1994 and by 1999 a total of 103 ex-patients had been rehoused in these community hostels. This effort was facilitated by the extension of the psychiatric reform programme and the community interventions for the acceptance of ex-patients by the islanders. Accommodation and living conditions were then upgraded and the buildings were repaired. A number of psychosocial interventions addressed the crucial issues of patients' rights, relationships and social roles. The interventions applied to all patients without exception and also involved patients' families, care staff, the institution and the local community. The evaluation study of a 3-year pilot intervention implemented at Leros Patriot Institution for Social Care and Welfare, the so-called Leros PIKPA, for people with severe intellectual disabilities reported improved resident care and adaptive behaviour, increased communication between residents and families, and positive changes in resident-management practices and attitudes towards people with disabilities post-intervention.^6^ Additionally, asylum administration and the local community were sensitised to residents' needs. In a study examining the quality of life of ex-patients residing in community hostels on Leros, the majority showed a very low level of psychopathology, but poor levels of social activity and community skills.^7^ Also, they displayed institutional behaviour and had largely lost the skills necessary for living independently in society. However, they reported high levels of satisfaction for their present living situation in the community hostels, their work, and the feeling of safety in the community, but low levels of satisfaction for their relationships with family members. During the developmental phase of the first psychiatric reform programme, Leros I, several positive outcomes were achieved via the implementation of time-limited projects, new policies and substantial financial and technical assistance from the EU. These outcomes included reduction of the number of psychiatric beds, development of 250 new services and programmes, such as community mental health services (mental health centres, day centres and supported residential services), reduction of the average length of stay in mental hospitals, and increase in staff numbers.^8^

It was, however, the Psychargos programme that advanced developments for community-based services.^9,10^ The programme, with funding of 700 million euro, started initially as a 10-year project (from 1997 to 2006). Its aims included: (a) completing the psychiatric reforms, with the deinstitutionalisation of the remaining long-term psychiatric patients in the eight mental hospitals and their closure; (b) the resettlement of ex-patients into the newly established psychosocial rehabilitation and housing services; and (c) further development of the network of community mental health services. In 1999, the most progressive Mental Health Law 2716 was passed.^11^ It set the basic principles of mental health practice in Greece, identified the ‘units of mental health’ and introduced the concept of ‘social cooperative units’, which would provide people with mental illness the opportunity to work and ideally live off this work. Unfortunately, in 1999 the strong Athens earthquake seriously damaged a big part of the existing long-stay psychiatric hospital. The initial plan of the Psychargos programme was then reviewed and eventually implemented in two phases.

The first phase (Psychargos I) lasted from 1997 to 2001 and included training of mental health professionals, improving infrastructure and residents' daily living, and preparing patients for community living through employment skills training. The second phase (Psychargos II revised) lasted from 2001 to 2010 with the continued aim of deinstitutionalisation and the development of community-based mental health services.^9,12–16^ The closure of mental health hospitals, the establishment of psychiatric services in general hospitals, and the development or expansion of specialist services were some of its main targets (Table 1). The sectorisation of mental health services, with care coordination and delivery in relatively small, discrete geographical areas across the country, was another main target of the reform.

**Table 1:** Mental health services developed under the Psychargos programme

Type of service	*N*	Beds/places
Community mental health centres	43	

Day centres	57	855–900 places

Mobile units	25	

Out-patient psychiatric clinics	37	

Psychiatric departments in general hospitals	28	650 beds

Hostels and sheltered apartments	343	3100 beds and 2000 places in rehabilitation services

Until now, four mental health hospitals have been closed down, retaining only administrative facilities to serve the hostels, out-patient units and rehabilitation services in their area. The remaining hospitals provide services to the several hostels, out-patient facilities and social rehabilitation units in their areas, and the numbers of patients with chronic illness has substantially decreased. Also, a number of non-governmental organisations (NGOs) were set up for the completion of the Psychargos projects. In 2012, 65 NGOs were involved with 220 units (30% of the total mental health units) covering 50% of the deinstitutionalisation beds and a total budget of 45 million euro in 2010. With respect to the private sector, there were 4207 in-patient beds in 2007.^8,11^ The last revision of the programme covers the period of 2011–2020.

In an *ex post* evaluation, focus groups of service providers and service users reported several positive elements of the Psychargos programme reforms, including a widespread reduction of hospital-based long-stay accommodation, a complete closure of some mental hospitals, and a large number of community services established in many parts of the country.^10^ Moreover, they acknowledged that local communities became gradually more accepting of people with mental illness and that there were also positive changes in the attitudes of staff towards person-centred care. However, both focus groups commented on the fragmented nature of the reforms. The main concerns included: a marked lack of coordination; inadequate provision on the ground; lack of thoughtful planning and implementation; inequity in the development of services between different areas around the country; lack of information about locally available services and poor information flow between different services; no quality assurance mechanisms or systems for clinical governance; and a paucity of monitoring systems. The focus groups also identified important gaps in specialist mental health services, such as those for children and adolescents.

Indeed, a major target of the psychiatric reform in Greece was the closure of the huge Child Psychiatric Hospital of Attica, which had been operating since 1960. Its patients, mainly children with severe intellectual disabilities, were moved to community facilities. The development of psychiatric services for children has followed a different course than that for adults as only 30% of the scheduled child guidance clinics have been created.^11,17^ Also, the distribution of child psychiatry services has been uneven in favour of the Athens area, with some great geographical areas lacking a single child psychiatry service. At the same time, the Greek financial crisis in recent years has had a very negative impact on the provision of child psychiatric services. The existing child and adolescent mental health services in the national healthcare system now operate with 10–40% fewer employees, whose salaries have been cut by 40% and who are not paid regularly. A large portion of the more experienced personnel has been forced into retirement. The demand for public services has increased considerably, since psychopathology of children and adolescents has risen as an effect of the crisis on the family as a whole, and family finances do not allow continuation of treatment in the private sector. A recent survey in both public and private child psychiatric institutions compared data from 2007 and 2011 (2 years before and 2 years after the implementation of austerity measures). Findings revealed a 39.8% increase in new cases in public out-patient services for children and 25.5% for adolescents, while percentages have dropped by a total of 35.4% in the private sector. As a result, in most public child and adolescent mental health services the waiting time for ordinary cases has tripled and is now longer than a month, while in special cases it can be up to 1 year.^18,19^

To summarise, deinstitutionalisation and development of community mental health services have advanced significantly since the 1980s, when psychiatric reform started in Greece. However, this reform is still incomplete, given that sectorisation, adequate primary care policies, inter-sectoral coordination and specialised services – such as those for children and adolescents, and for people with autism and intellectual disabilities, and geriatric and forensic services – are under-developed. This problematic situation is further complicated by the severe impact of the current financial crisis that has led to increased demand for services, funding difficulties and staff shortages. For instance, according to the Ministry of Health Directorate for Mental Health, patient visits to emergency units, outpatient departments and mental health clinics in the national healthcare general hospitals have increased by 120% during 2011–2013.^18^ Unemployment and low income were found to be significantly correlated with visits to outpatient departments and emergency units.^20^ Preserving the existing services, optimal usage of the available limited resources and linking funding to service performance are essential prerequisites to the completion of the psychiatric reform in Greece.
